# *Aspergillus* section *Flavi* community structure in Zambia influences aflatoxin contamination of maize and groundnut

**DOI:** 10.1016/j.ijfoodmicro.2017.08.014

**Published:** 2017-11-16

**Authors:** Paul W. Kachapulula, Juliet Akello, Ranajit Bandyopadhyay, Peter J. Cotty

**Affiliations:** a School of Plant Sciences, University of Arizona, Tucson, AZ 85721, USA; b School of Agricultural Sciences, University of Zambia, P.O Box 32379, Lusaka, Zambia; c International Institute of Tropical Agriculture (IITA), Lusaka, Zambia; d International Institute of Tropical Agriculture (IITA), PMB 5320 Ibadan, Nigeria; e USDA-ARS, School of Plant Sciences, University of Arizona, Tucson, AZ 85721, USA

**Keywords:** *Aspergillus*, *Flavi*, Aflatoxin, Maize, Groundnuts, Zambia

## Abstract

Aflatoxins are cancer-causing, immuno-suppressive mycotoxins that frequently contaminate important staples in Zambia including maize and groundnut. Several species within *Aspergillus* section *Flavi* have been implicated as causal agents of aflatoxin contamination in Africa. However, *Aspergillus* populations associated with aflatoxin contamination in Zambia have not been adequately detailed. Most of Zambia's arable land is non-cultivated and *Aspergillus* communities in crops may originate in non-cultivated soil. However, relationships between *Aspergillus* populations on crops and those resident in non-cultivated soils have not been explored. Because characterization of similar fungal populations outside of Zambia have resulted in strategies to prevent aflatoxins, the current study sought to improve understanding of fungal communities in cultivated and non-cultivated soils and in crops. Crops (*n* = 412) and soils from cultivated (*n* = 160) and non-cultivated land (*n* = 60) were assayed for *Aspergillus* section *Flavi* from 2012 to 2016. The L-strain morphotype of *Aspergillus flavus* and *A. parasiticus* were dominant on maize and groundnut (60% and 42% of *Aspergillus* section *Flavi*, respectively). Incidences of *A. flavus* L-morphotype were negatively correlated with aflatoxin in groundnut (log y = 2.4990935 − 0.09966x, R^2^ = 0.79, *P* = 0.001) but not in maize. Incidences of *A. parasiticus* partially explained groundnut aflatoxin concentrations in all agroecologies and maize aflatoxin in agroecology III (log y = 0.1956034 + 0.510379x, R^2^ = 0.57, *P* < 0.001) supporting *A. parasiticus* as the dominant etiologic agent of aflatoxin contamination in Zambia. Communities in both non-cultivated and cultivated soils were dominated by *A. parasiticus* (69% and 58%, respectively). *Aspergillus parasiticus* from cultivated and non-cultivated land produced statistically similar concentrations of aflatoxins. Aflatoxin-producers causing contamination of crops in Zambia may be native and, originate from non-cultivated areas, and not be introduced with non-native crops such as maize and groundnut. Non-cultivated land may be an important reservoir from which aflatoxin-producers are repeatedly introduced to cultivated areas. The potential of atoxigenic members of the *A. flavus*-L morphotype for management of aflatoxin in Zambia is also suggested. Characterization of the causal agents of aflatoxin contamination in agroecologies across Zambia gives support for modifying fungal community structure to reduce the aflatoxin-producing potential.

## Highlights

• The chief etiologic agent of aflatoxin contamination in Zambia is *A. parasiticus*.
• Crop aflatoxin decreases with increase in frequencies of the *A. flavus* L morphotype.
• Cultivated and non-cultivated areas have similar frequencies of aflatoxin-producers.
• Fungi in non-cultivated areas may influence crop aflatoxin contamination.

## 1 Introduction

Maize and groundnut are important crops for both commercial and smallholder farmers in Zambia. Maize is cultivated by > 80% of the farmers in all agroecologies for self-consumption, sale or both (Tembo and Sitko, 2013) and contributes up to 50% of daily calorie intake (FAO, 2014). Groundnut is the second most widely cultivated crop and is grown in all the agroecologies of Zambia (Tembo and Sitko, 2013). International demand for groundnut provides an important potential source of income. Groundnut and maize are susceptible to aflatoxin contamination and heavy dependence on these two crops in Zambia may result in significant aflatoxin associated hazards.

Consumption of aflatoxin-contaminated food may cause cirrhosis, liver cancer, stunting, reduced immunity, reduced weight-gain and/or rapid death (Gong et al., 2004, Lewis et al., 2005, Liu et al., 2012, Probst et al., 2007, Reddy and Raghavender, 2007, Turner et al., 2003, Williams et al., 2004). Enforcement of regulatory limits on aflatoxin concentrations in foods and feeds causes loss of markets for agricultural products and reduced income (van Egmond et al., 2007, Wu, 2014). Europe and South Africa, with regulatory limits of 4 and 10 ppb total aflatoxin, respectively, have been important markets for agricultural commodities from Zambia. The country exported over 8000 metric tons of groundnut to Europe in the 1960s. However, this market collapsed due in part to enforcement of aflatoxin regulations in Europe (Sitko et al., 2011). Improved knowledge of the etiology of aflatoxin contamination in Zambia may reveal management options (Cotty et al., 2008).

Aflatoxin contamination is caused by crop infection by one or more species in *Aspergillus* section *Flavi*. The fungi disperse from soil, organic matter, and alternative hosts to developing crops. Crop infection and subsequent aflatoxin production are high when conditions are hot and dry during crop development and warm and humid after crop maturation and/or harvest (Cotty and Jaime-Garcia, 2007). The species most notorious for crop contamination are *Aspergillus flavus* (produces only B aflatoxins), *A. parasiticus* (produces both B and G aflatoxins) and two unnamed taxa S_B_ (only B aflatoxins) and S_BG_ (both B and G aflatoxins; Cotty et al., 2008, Probst et al., 2010). Aflatoxin-producers are often sorted on the basis of sclerotial morphology (Cotty, 1989). L morphotype fungi produce few large sclerotia (average diameter > 400 μm) and S morphotype fungi produce numerous small sclerotia (average diameter < 400 μm; (Cotty, 1989). Fungi with S morphology frequently produce large quantities of aflatoxins. Molecular phylogenetic studies suggest S morphotype aflatoxin-producers are actually several species: a) *A. flavus* S strain; b) Lethal Aflatoxicosis Fungus (LAF) S_B_ that severely contaminated maize and led to many deaths in Kenya (Probst et al., 2007); c) the un-named taxon S_BG_ from West Africa (Cotty and Cardwell, 1999); and d) *A. minisclerotigenes* (Pildain et al., 2008). *Aspergillus parasiticus* is also frequently described as an etiologic agent of groundnut aflatoxin contamination (Horn and Dorner, 1998). Although all of these aflatoxin-producers may cause dangerous aflatoxin levels in crops when present in a conducive environment, genotypes vary in average aflatoxin-producing potential and the relative importance of specific etiologic agents may vary from one region to another (Cotty et al., 2008). Frequencies of aflatoxin-producers on crops and relationships of fungal communities in non-cultivated soils to those resident in cultivated soils have not been characterized in Zambia. Non-cultivated areas, such as forests, may be reservoirs for aflatoxin-producers that may either move into cropping systems or cause contamination of non-cultivated fruits and grains (Boyd and Cotty, 2001). Potential causal agents of aflatoxin contamination in cultivated and non-cultivated plants in Zambia need characterization, and the relationship of fungal community structure to aflatoxins in groundnut and maize needs investigation (Kachapulula et al., 2017).

In order to explore possibilities for limiting aflatoxin contamination in Zambia, compositions of *Aspergillus* section *Flavi* communities associated with aflatoxin contamination infecting maize and groundnut were explored and aflatoxin production by these communities was characterized and related to *Aspergillus* section *Flavi* resident in non-cultivated areas. *Aspergillus parasiticus* was found to be an important etiologic agent for both maize and groundnut and communities of *Aspergillus* section *Flavi* resident in native, non-cultivated areas appear to have influenced compositions of fungi infecting crops.

## 2 Methods

### 2.1 Study area

Zambia lies between 8° and 18° South, and 22° and 34° East of the Greenwich meridian and has three agroecologies designated I, II, and III (Bunyolo et al., 1995). Agroecology III is the northern most with elevation 1100 to 1700 masl, annual rainfall > 1000 mm, and average annual temperature, 30–33 °C (Bunyolo et al., 1995). Agroecology II covers most of the land in agricultural production and all of central Zambia. Elevation extends from 900 to 1300 masl with 800–1000 mm annual rain, and 30–32 °C average annual temperature. Agroecology I extends across southern Zambia with elevations below 900 masl, < 800 mm average annual rainfall, and 30–36 °C average annual temperature (Bunyolo et al., 1995).

### 2.2 Sampling

**Fig. 1:**
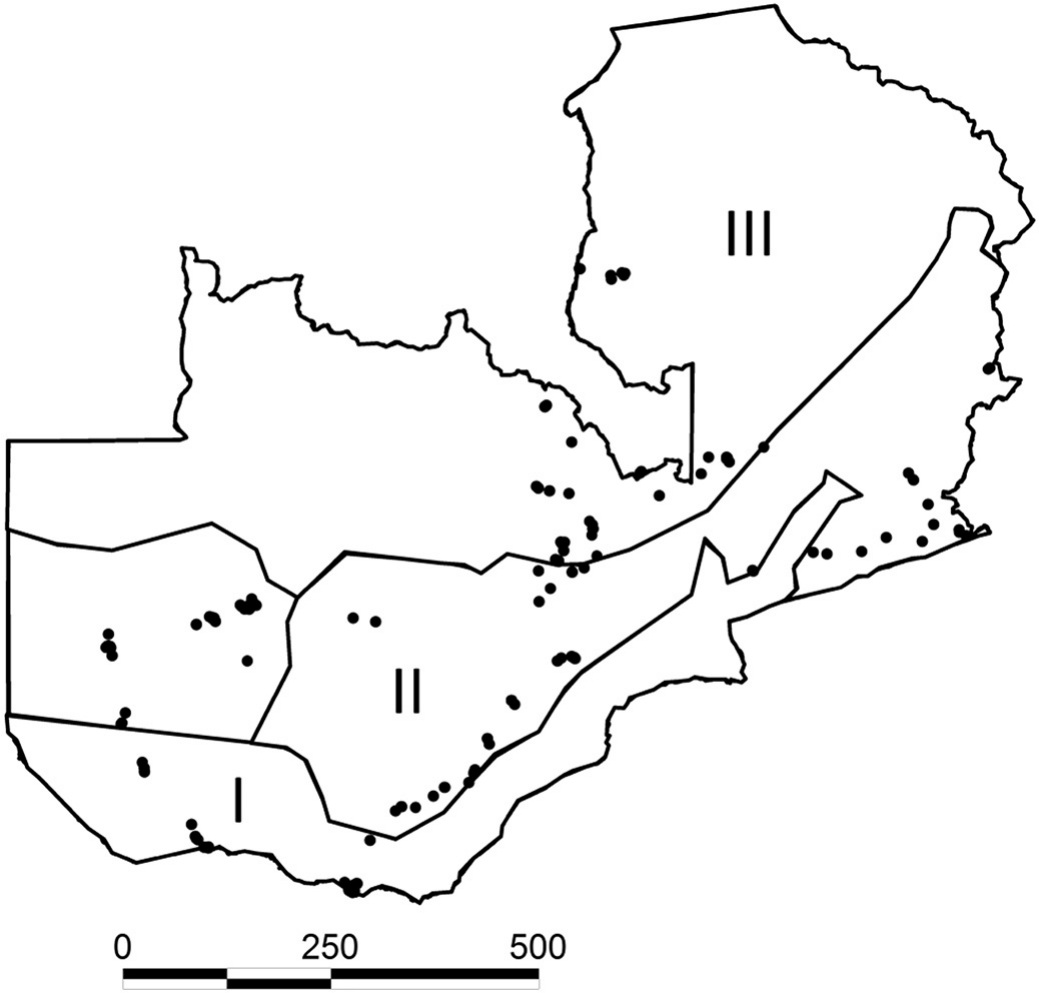
Map of the three agroecologies of Zambia (I, II, and III). Filled circles indicate locations from which maize and groundnut samples were collected. Scale bar is in kilometers.

Maize (*n* = 250) and groundnut (*n* = 162) samples from a previous study (Kachapulula et al., 2017) representing 27 districts and all three agroecologies of Zambia (Table 1, Table 2) were included in the current study. In addition, 220 soils were sampled from cultivated fields (*n* = 160) and from non-cultivated areas (*n* = 60), in 16 districts covering all three agroecologies (Fig. 1). Briefly, at least 4 locations or fields were sampled in each district. Three composite soil samples (100–175 g each) were obtained from each field by scooping soil subsamples at three random locations in each field to a depth of 2 cm (Cotty, 1997). In each agroecology, crop and soil samples were collected during the same trip with sampling occurring during January and May (agroecologies 1, 2, & 3), and November (agroecologies 1 & 2). Soil and crop samples were dried in a forced air oven (40 °C) to 5–8% water content to prevent fungal growth after receipt and sealed in plastic bags to prevent rehydration. All crop and soil samples were imported to the USDA, ARS. Laboratory in the School of Plant Sciences, University of Arizona under permit number P526P-12-00853 awarded to Peter J. Cotty by the Animal Plant Health Inspection Service of USDA.

(Redrawn from Kachapulula et al., 2017).

**Table 1:** Distribution of fungi of *Aspergillus* section *Flavi* on maize†.

Agroecology	District	# of isolates	% L⁎	% S	% P	% T	CFU/g
III	Mansa	494	88	5	7	0	603
	Mpongwe	33	93	7	0	0	27
	Average††		91^a(x)^	6^a(y)^	4^b(y)^	0^a(y)^	315
II	Choma	111	20	24	56	0	12
	Kabwe	125	31	22	47	0	13
	Kalomo	95	52	6	42	0	2080
	Kaoma	244	61	18	15	6	843
	Kapiri-mposhi	148	72	0	28	0	13
	Mazabuka	70	6	70	24	0	41,167
	Mongu	180	79	2	19	0	37
	Monze	92	0	40	60	0	126
	Senanga	152	73	14	13	0	626
	Average††		44^b(x)^	22^a(x)^	34^a(x)^	1^a(y)^	4991
I	Livingstone	68	30	22	48	0	146
	Sesheke	150	59	21	20	0	686,602
	Average††		45^b(x)^	22^a(xy)^	34^a(xy)^	0^a(y)^	343,374
Across agroecology			60^(x)^	16^(y)^	24^(xy)^	0^(z)^	

⁎ L, S, P and T represent *A. flavus* L-morphotype, S-morphotype fungi, *A. parasiticus* and *A. tamarii*, respectively.

† Percent data were arcsine transformed and CFU/g data were log transformed prior to analyses. Values followed by the same letter in each column (a, b, c) or row (x, y, z) do not differ by Tukey's HSD test (α = 0.05).

†† Average percentages for locations in each district were used for analyses and only district averages are presented.

**Table 2:** Distribution of fungi of *Aspergillus* section *Flavi* on groundnut†.

Agroecology	District	# of isolates	% L⁎	% S	% P	% T	CFU/g
III	Mansa	359	50	19	31	0	113
	Mpongwe	53	20	14	66	0	27
	Average††		35^a(xy)^	17^b(y)^	49^a(x)^	0^a(z)^	70
II	Choma	98	0	53	47	0	12,572
	Kabwe	126	20	44	36	0	576
	Kalomo	88	0	44	56	0	590
	Kaoma	374	8	43	48	1	521
	Kapiri-mposhi	99	1	34	65	0	362
	Mazabuka	81	0	64	36	0	7806
	Mongu	353	27	43	30	0	48,098
	Monze	123	0	57	43	0	32,697
	Senanga	124	4	65	31	0	110
	Average††		7^b(y)^	50^a(x)^	44^a(x)^	0^a(y)^	11,481
I	Livingstone	101	51	43	6	0	7926
	Sesheke	158	19	19	60	2	70
	Average††		35^a(x)^	31^b(x)^	33^a(x)^	1^a(y)^	3998
Across agroecology			26^(y)^	32^(x)^	42^(x)^	0^(z)^	

⁎ L, S, P and T represent *A. flavus* L-morphotype, S-morphotype fungi, *A. parasiticus* and *A. tamarii*, respectively.

† Percent data were arcsine transformed prior to analyses. Values followed by the same letter in each column (a, b, c) or row (x, y, z) do not differ by Tukey's HSD test (α = 0.05).

†† Average percentages for locations in each district were used for analyses and only district averages are presented.

### 2.3 Isolation and identification of fungi from maize, groundnut and soils

Maize and groundnut samples were ground in a knife mill (Grindomix GM200, Retsch GmbH, Haan, Germany) to pass a #12 sieve, and homogenized. Fungi were recovered from ground crop material and dry soil using dilution plate technique on modified rose Bengal agar (Cotty, 1994). Briefly, ground crop material and soil (0.1 to 10 g) were shaken in 50 ml sterile distilled water (20 min, 100 rpm) on a reciprocal shaker (KS-501, IKA Works Inc., Wilmington, NC, USA). Dilution plating was performed on modified rose Bengal agar in triplicate. Plates were incubated (3 days, 31 °C, dark) and up to eight colonies of *Aspergillus* section *Flavi* were transferred to 5-2 agar (5% V8 Vegetable Juice (Campbell's Soup Company, Camden, N.J., USA); 2% agar, pH 5.2, Cotty, 1989). Fungi were stored in sterile water (2 ml) as plugs of sporulating culture after incubation for 7 days at 31 °C (Cotty, 1988). Isolations were performed at least twice from each sample. *Aspergillus* species and strains were identified using both macroscopic and microscopic characteristics (Cotty, 1989, Cotty, 1994, Klich and Pitt, 1988, Probst et al., 2007).

### 2.4 Community composition of *Aspergillus* section *Flavi* from soils of cultivated and non-cultivated areas

Quantities and community composition of *Aspergillus* section *Flavi* from cultivated and non-cultivated areas were compared. The total quantity of section *Flavi* fungi from each crop and soil sample was calculated as Colony Forming Units (CFU) per gram. Community composition of section *Flavi* was described as percent of *A. flavus* L-morphotype (Cotty, 1989), undelineated S-morphotype species (Probst et al., 2007), *A. parasiticus*, and *A. tamarii* recovered from each sample. Quantities of section *Flavi* members were calculated as the percent detected during isolation multiplied by total section *Flavi* CFU/g.

### 2.5 Aflatoxin producing potential of *A. parasiticus* from cultivated fields and non-cultivated areas

*Aspergillus parasiticus* isolates from maize (6), groundnut (6), and either cultivated (16) or non-cultivated soil (35) were assayed for aflatoxin-producing potential on sterile maize and groundnut. Fungi were inoculated onto undamaged, sterile maize and groundnut kernels (10 g/250 ml Erlenmeyer flask) previously autoclaved for 60 min, cooled to room temperature, and adjusted to 30% water content. The following steps were performed to adjust moisture content of the maize and groundnut kernels post-autoclaving and to inoculate: (1) The initial moisture after autoclaving was measured using an HB43 Halogen Moisture Analyzer (Mettler Toledo, Columbus, OH) and the amount of water needed to raise the moisture of kernels to 30% determined; (2) Conidia of each isolate from 7-day-old cultures (grown on 5% V8-juice; 2% agar, pH 5.2, Cotty, 1989) were harvested into sterile, deionized water (10 ml); (3) concentrations of conidia were estimated with turbidity (Orbeco-Hellige turbidimeter TB300IR; Orbeco Analytical Systems, Farmingdale, NY) and using a nephelometric turbidity unit (NTU)-versus-CFU standard curve, where y is equal to 49,937x (x is NTU, and y is conidia/ml) (Probst et al., 2010); (4) A spore suspension containing 1 × 10^6^ conidia (usually about 500 μl) was mixed with water to bring the final volume to that determined in step 1 above, added to 10 g of kernels in a flask and swirled to coat the kernels. Inoculated grains were incubated (7 days, 100% RH, 31 °C). After incubation, sample cultures were blended in 50 ml of 70% methanol. The slurry was allowed to separate for 30 min and the supernatant was spotted directly onto thin-layer chromatography (TLC) plates (Silica gel 60, EMD, Darmstadt, Germany) adjacent to aflatoxin standards (Aflatoxin Mix Kit-M, Supelco) containing known quantities of aflatoxins B_1_, B_2_, G_1_ and G_2_. Plates were developed in ethyl ether-methanol-water, 96:3:1, air-dried and aflatoxins visualized under 365-nm UV light. Aflatoxins were quantified directly on TLC plates using a scanning densitometer (TLC Scanner 3, Camag Scientific Inc., Wilmington, N.C.).

Five market samples found in a previous study to contain > 500 ppb total aflatoxins were subjected to aflatoxin analyses by TLC shortly after grinding to evaluate presence of B and G aflatoxins. Fifty grams of ground crop were extracted with 70% methanol (250 ml) and the methanol extract was directly spotted onto TLC plates and separated and quantified as above. The specific aflatoxins were identified by comparison with standards spotted on the same plate.

### 2.6 Data analysis

Statistical analyses were performed with JMP 11.1.1 (SAS Institute, Cary, NC). Means for fungal frequencies in cultivated and adjacent non-cultivated soils in each district were compared using paired *t*-test were compared using the paired *t*-test and multiple comparisons were performed with Analysis of Variance (general linear models) followed by mean separation with Tukey's HSD test. Relationships between crop aflatoxin concentration and quantities of each member of *Aspergillus* section *Flavi* were investigated with regression analyses. Data were tested for normality and, if required, log (for aflatoxin concentrations and propagules per gram) or arcsine (for percentages) transformed to normalize distributions. Actual means are presented for clarity. All tests were performed at α = 0.05.

## 3 Results

### 3.1 Fungi in maize and groundnuts

*Aspergillus* section *Flavi* was recovered from all maize and groundnut samples. A total of 4099 isolates were characterized from 412 samples (Table 1, Table 2). The frequencies of occurrence of *Aspergillus* section *Flavi* members in maize differed significantly (ANOVA, F_3,48_ = 18.6842, *P* < 0.001) across agroecologies, with the *A. flavus* L-morphotype dominating communities (60%), followed by *A. parasiticus* (24%) and S-morphotype fungi (16%, Table 1). In all agroecologies, the *A. flavus* L-morphotype was the most common member of *Aspergillus* section *Flavi* on maize making up 91%, 44%, and 45% of section *Flavi* in agroecologies III, II, and I, respectively. L morphotype frequencies in region III were higher than either region II or I (Tukey's HSD, *P* < 0.05). *Aspergillus parasiticus* and fungi with S morphology were more common in agroecologies II and I (agroecology averages = 22 to 34% of section *Flavi*) than in agroecology III (average 4 to 6%).

In groundnut, there were also significant differences in *Aspergillus* section *Flavi* across agroecologies (ANOVA, F_3,48_ = 36.6726, *P* < 0.001), with *A. parasiticus* dominating (42%), then S-morphotype fungi (32%) and *A. flavus* L strain morphotype (26%, Table 2). *Aspergillus flavus* L strain morphotype frequencies on groundnut were higher in agroecologies III (35%) and I (35%) than II (7%, Tukey's HSD, *P* = 0.021) whereas the S-morphotype was more common in agroecology II (50%) than in I (31%) and III (17%, Tukey's HSD *P* = 0.0048). *Aspergillus parasiticus* was equally prevalent on groundnut from all agroecologies (*P* = 0.5512, Tukey's HSD, Table 2).

Frequencies of section *Flavi* members differed between maize and groundnut (Table 3) with the *A. flavus* L-strain morphotype higher (t_103_ = 8.468044, *P* < 0.001) in maize (60%) than groundnut (26%, Table 3) and *A. parasiticus* higher (t_103_ = 3.97205, *P* < 0.001) in groundnut (42%) than maize (24%; Table 3). Fungi with S morphology followed the same trend as *A. parasiticus* (Table 3). In agroecology I, each section *Flavi* member occurred at similar frequency on maize and groundnut (Table 3).

**Table 3:** Incidence of *A. flavus* L strain morphotype, S strain morphotype fungi, and *A. parasiticus* on maize and groundnut in three agroecologies of Zambia.

Agroecology	% L†		% S		% P	
	Maize	Groundnut	Maize	Groundnut	Maize	Groundnut
III	91⁎^x^	35^x^	6⁎^x^	17^y^	4⁎^y^	49^x^
II	44⁎^y^	7^y^	22⁎^x^	50^x^	34⁎^x^	44^x^
I	45^y^	35^x^	22^x^	31^y^	34^x^	33^x^

Percent data were arcsine transformed before analyses. Values followed by the same letter (x/y) within the same column do not differ by Tukey's HSD (α = 0.05).

† L, S, and P represent *A. flavus* L morphotype, S morphotype fungi and *A. parasiticus*, respectively.

⁎ Maize and groundnut values in the same fungus differ by paired *t*-test (α = 0.05).

### 3.2 Association between quantity of *Aspergillus* section *Flavi* and aflatoxin concentration

Quantities (CFU/g) of the *A. flavus* L strain morphotype were inversely related to aflatoxin concentration in groundnut from agroecology I (log y = 2.4990935 − 0.09966x, R^2^ = 0.79, *P* = 0.001), but were not related to either groundnut or maize aflatoxin concentrations in the other agroecologies (Table 4). Quantities of S-morphology fungi increased with aflatoxin concentrations in maize only from agroecology II (log y = 1.2273858 + 0.243253x, R^2^ = 0.37, *P* < 0.001). *Aspergillus parasiticus* quantities were predictive of aflatoxin concentrations in groundnut in all three agroecologies (agroecology I, log y = 1.9957586 + 0.1323517x, R^2^ = 0.63, *P* = 0.018; agroecology II, log y = 0.4673417 + 0.3513556x, R^2^ = 0.30, *P* < 0.001; agroecology III, log y = 0.25685 + 0.2277388x, R^2^ = 0.24, *P* = 0.0491) and in maize from agroecology III (log y = 0.1956034 + 0.510379x, R^2^ = 0.57, *P* < 0.001).

**Table 4:** Coefficients of determination and other parameters for regression analyses of relationships between crop aflatoxin concentration and the quantity of propagules of *A. parasiticus*, the L morphotype of *A. flavus*, and S morphotype fungi.

Agroecology	Community component⁎ (Quantity of)	Intercept	Rate of increase^X^	Coefficient of determination (R^2^)	Model significance (*P*)^Y^
I	Groundnut				
	Quantity of L (CFU/g)	2.50	− 0.10	0.79	0.001
	Quantity of P (CFU/g)	1.9957586	0.13	0.63	0.018
	Quantity of S (CFU/g)	NS^Z^	NS	NS	NS
	Maize				
	Quantity of L (CFU/g)	NS	NS	NS	NS
	Quantity of P (CFU/g)	NS	NS	NS	NS
	Quantity of S (CFU/g)	NS	NS	NS	NS
II	Groundnut				
	Quantity of L (CFU/g)	NS	NS	NS	NS
	Quantity of P (CFU/g)	0.4673417	0.35	0.3	< 0.001
	Quantity of S (CFU/g)	NS	NS	NS	NS
	Maize				
	Quantity of L (CFU/g)	NS	NS	NS	NS
	Quantity of P (CFU/g)	NS	NS	NS	NS
	Quantity of S (CFU/g)	1.23	0.24	0.37	< 0.001
III	Groundnut				
	Quantity of L (CFU/g)	NS	NS	NS	NS
	Quantity of P (CFU/g)	0.26	0.23	0.24	0.049
	Quantity of S (CFU/g)	NS	NS	NS	NS
	Maize				
	Quantity of L (CFU/g)	NS	NS	NS	NS
	Quantity of P (CFU/g)	0.20	0.51	0.57	< 0.001
	Quantity of S (CFU/g)	NS	NS	NS	NS

⁎ L, P and S represent *A. flavus* L-morphotype, *A. parasiticus* and S-morphotype fungi, respectively. CFU/g data was log-transformed prior to analyses.

X This value represents the change in aflatoxin for a unit change in CFU/g of crop. Negative values reflect lowering aflatoxin concentrations associated with increased quantities of fungus.

Y Significance set at *P* = 0.05.

Z NS = non-significant.

### 3.3 *Aspergillus* section *Flavi* from cultivated and non-cultivated soils

A total of 2128 *Aspergillus* section *Flavi* isolates were obtained from 220 soil samples (Table 5). One or more *Aspergillus* section *Flavi* species was recovered from all soils. Species differed in overall frequency in both un-cultivated (F_3,168_ = 101.2705, *P* < 0.001) and cultivated (F_3,324_ = 113.0661, *P* < 0.001) soils (Table 5). *Aspergillus parasiticus* was the most frequent *Aspergillus* section *Flavi* species in both soil types (58% in cultivated and 69% in non-cultivated soils), and in all agroecologies, with the exception of cultivated soils in agroecology I where *A. flavus* L-strain morphotype was most frequent (Table 5). *Aspergillus parasiticus* was most frequent in the coolest agroecology, agroecology III, and, overall, least frequent in the agroecology with the highest average temperature and the least rainfall, agroecology I (Table 5). The frequency of *Aspergillus* section *Flavi* with S morphology did not differ among agroecologies in both cultivated (ANOVA, F_2,79_ = 2.7790 *P* = 0.0682) and non-cultivated soils (ANOVA, F_2,40_ = 0.3660, *P* = 0.6958, Table 5).

**Table 5:** Distribution of fungi of *Aspergillus* section *Flavi* in non-cultivated and cultivated soils from three agroecologies§.

Agroecology	Samples (#)	Isolates (#)	NC†				CV†				CFU/g	Temp.†† (°C)	Rain†† (mm)
			% L⁎	% S	% P	% T	% L	% S	% P	% T			
III	46	554	7^b(y)^	6^a(y)^	86^a(x)^	1^a(y)^	4^b(y)^	8^a(y)^	88^a(x)^	0^a(y)^	85	30–33	> 1000
II	152	1280	32^ab(y)^	3^a(y)^	57^b(x)^	8^a(y)^	9^b(yz)^	22^a(y)^	66^b(x)^	3^a(z)^	107	30–33	800–1000
I	21	294	33^a(y)^	3^a(y)^	64^ab(x)^	0^a(y)^	68^a(x)^	12^a(y)^	19^c(y)^	1^a(y)^	108	30–36	< 800
Across agroecologies	73	709	24^(y)^	4^(yz)^	69^(x)^	3^(z)^	27^(y)^	14^(y)^	58^(x)^	1^(z)^	100		

⁎ L, S, P and T represent *A. flavus* L-morphotype, S-morphotype fungi, *A. parasiticus* and *A. tamarii*, respectively.

† NC = non-cultivated soil, CV = cultivated soil.

†† Temp. = average annual temperature and rain = average annual rainfall.

§ Percent data were arcsine transformed prior to analyses. L, S, P, T indicate *A. flavus* L morphotype, S morphotype fungi, *A. parasiticus* and, *A. tamarii*, respectively. Values followed by the same letter in each column (a, b, c) or row (x, y, z) for non-cultivated and for cultivated soils, do not differ by Tukey's HSD test (α = 0.05). Districts sampled include Mansa, Mpongwe and Kitwe in agroecology III; Chibombo, Chipata, Chongwe, Kabwe, Kaoma, Kapiri-mposhi, Lundazi, Mkushi, Petauke, Senanga and Serenje in agroecology II; Livingstone and Sesheke in agroecology I.

Frequencies of the *A. flavus* L-strain morphotype, S strain morphotype fungi, and *A. parasiticus* did not differ significantly (*P* > 0.05, paired *t*-test) between cultivated and non-cultivated soils regardless of agroecology. However, the overall quantity of *Aspergillus* section *Flavi* (CFU/g) was higher (*P* < 0.001) in cultivated (175 CFU/g) than non-cultivated soils (25 CFU/g, Table 6).

**Table 6:** Fungi of *Aspergillus* section *Flavi* in non-cultivated and cultivated soils^a^.

District	# of samples	# of isolates	% *A. flavus*-L		% S-morphotype		% *A. parasiticus*		% *A. tamarii*		CFU/g	
			NC^c^	CV^c^	NC	CV	NC	CV	NC	CV	NC	CV
Mansa	24	338	2	0	0	0	96	100	2	0	33	82
Mpongwe	23	312	12	7	13	12	75	81	0	0	11	179
Chibombo	23	275	0	11	0	46	100	43	0	0	30	511
Chongwe	21	208	23	28	0	1	59	67	18	4	5	101
Kaoma	30	277	76	2	2	8	7	65	15	25	19	20
Senanga	24	273	28	8	10	19	62	73	0	0	10	182
Sesheke	21	294	33	68	3	12	64	19	0	1	65	150
Average	24	281	25	18	4	14	66	64	5	4	25^b^	175^b^

a Percent data were arcsine transformed and CFU/g data were log transformed prior to statistical comparisons.

b Non-cultivated and cultivated values differ by paired *t*-test (α = 0.05).

c NC = non-cultivated soil, CV = cultivated soil.

### 3.4 Aflatoxin production by *A. parasiticus* from crops and soils

On groundnut at 20 °C, there were no significant differences (ANOVA, F_3,58_ = 0.8027, *P* = 0.4974, Table 7) in concentrations of aflatoxins produced by *A. parasiticus* isolates from maize (Mean = 123,810 μg/kg), groundnut (Mean = 196,997 μg/kg), cultivated (Mean = 145,613 μg/kg) and non-cultivated soil (Mean = 125,106 μg/kg). Similarly, the four groups of *A. parasiticus* did not differ in aflatoxin production at all other temperatures (25, 30 and 35 °C) even when maize was used as the substrate (Table 7). The highest concentrations of aflatoxin were produced on groundnut at 25 °C by *A. parasiticus* from maize (Mean = 214,321 μg/kg), groundnut (Mean = 215,669 μg/kg), cultivated (Mean = 199,214 μg/kg) and non-cultivated soil (Mean = 196,632 μg/kg) and the least were at 35 °C. A similar trend was observed when maize was used as substrate (Table 7). At 20 °C, higher concentrations of aflatoxins were produced on groundnut than maize by *A. parasiticus* isolates from maize (Paired *t*-test, t_5_ = 4.120746, *P* = 0.009), groundnut (Paired *t*-test, t_5_ = 2.961985, *P* = 0.0252), cultivated (Paired *t*-test, t_15_ = 2.838941, *P* = 0.0124) and non-cultivated soil (Paired *t*-test, t_34_ = 2.06039, *P* = 0.0473, Table 7). However, at the other temperatures, aflatoxin production on the two substrates was similar. Aflatoxin production by all isolates combined differed on both groundnut (ANOVA, F_3,12_ = 25.1034, *P* < 0.001) and maize (ANOVA, F_3,12_ = 22.7206, *P* < 0.001) at different temperatures (Table 7). Aflatoxin production on groundnut by all isolates was similar at 20 °C, 25 °C and 30 °C and lower at 35 °C (Tukey's HSD, *P* < 0.001), whereas on maize, higher concentrations were produced at 25 °C and 30 °C than 20 °C and 35 °C (Tukey's HSD, *P* < 0.001, Table 7). All five highly contaminated maize and groundnut market samples examined were found to contain both aflatoxins B and G (data not shown).

**Table 7:** Aflatoxin-producing potential of *A. parasiticus* from crops and from cultivated and non-cultivated soils.

Source of isolate	Type of aflatoxin	Aflatoxin at 20 °C		Aflatoxin at 25 °C		Aflatoxin at 30 °C		Aflatoxin at 35 °C	
		Groundnut	Maize	Groundnut	Maize	Groundnut	Maize	Groundnut	Maize
Maize	B1	60,700	26,300	106,500	72,200	73,900	108,300	48,700	64,000
	B2	700	1200	700	4200	2800	2900	16,200	300
	G1	62,000	25,000	106,700	96,700	36,400	47,500	11,000	8100
	G2	400	700	400	2800	1800	2000	8000	200
	Total	123,800⁎	53,200	214,300	175,900	114,900⁎	160,700	83,900	72,600
Groundnut	B1	33,500	33,000	40,400	53,800	73,100	77,800	21,000	34,500
	B2	0	700	0	800	0	100	200	0
	G1	163,500	73,400	175,300	130,700	68,600	71,600	7700	14,900
	G2	0	400	0	500	0	100	100	0
	Total	197,000⁎	107,500	215,700	185,800	131,700	149,600	29,000	49,400
Agricultural soil	B1	40,600	39,200	53,200	66,500	73,500	63,200	26,200	37,400
	B2	900	1100	3300	4000	8900	2100	1200	2300
	G1	103,500	65,300	140,500	125,900	79,100	66,800	7700	18,200
	G2	600	800	2200	2800	5900	1400	900	1600
	Total	145,600⁎	106,400	199,200	199,200	167,400	133,500	36,000	59,500
Uncultivated soil	B1	32,000	25,100	53,700	44,500	66,000	73,900	30,700	34,500
	B2	3500	5300	4600	3700	9400	5100	1700	2300
	G1	87,400	48,300	135,200	82,000	58,900	57,700	10,500	15,900
	G2	2200	3500	3100	2500	6800	3400	1200	1500
	Total	125,100⁎	82,200	196,600	132,700	141,100	140,100	44,100	54,200
All isolates	Total	147,800^A^⁎	87,300^Y^	206,459^A^	173,415^X^	141,131^A^	145,985^X^	48,244^B^	58,931^Y^

⁎ Maize and groundnut values (total aflatoxin) at the same temperature differ by paired *t*-test (α = 0.05). Means in each column (total aflatoxin) are not significantly different by ANOVA. Means followed by the same letter (A/B for groundnut and X/Y for maize) for “All isolates” at the different temperatures do not differ by Tukey-Kramer's HSD (α = 0.05). Data were log transformed prior to analyses.

## 4 Discussion

Dangers aflatoxins pose to human health, livestock productivity and trade are widely recognized (Gong et al., 2004, Lewis et al., 2005, Liu et al., 2012, Probst et al., 2007, Reddy and Raghavender, 2007, Turner et al., 2003, van Egmond et al., 2007, Williams et al., 2004, Wu, 2014). Recent deaths from consumption of highly contaminated food in Kenya (Lewis et al., 2005, Probst et al., 2007) and Tanzania have increased recognition of the need to understand the etiologic agents of aflatoxin contamination in many parts of sub-Saharan Africa and the world. Although *A. flavus*, *A. parasiticus*, and the two unnamed taxa (S_B_ and S_BG_) are associated with aflatoxin contamination of crops in warm parts of the world, the most important etiologic agents vary from one region to another (Cotty and Cardwell, 1999, Horn and Dorner, 1998, Probst et al., 2007). In addition, some aflatoxin-producers have also been recovered from non-cultivated areas (Boyd and Cotty, 2001) from which they may move into cropped areas, and provide region-specific influences on composition of communities of aflatoxin-producing fungi infecting and contaminating crops. The current study provides insights into compositions of communities of *Aspergillus* section *Flavi* in Zambia resident on maize, groundnut and soils. Compositions were found to influence observed aflatoxin concentrations and insights were developed into how communities of *Aspergillus* section *Flavi* from non-cultivated areas might shape those observed in cultivated areas and on crops.

### 4.1 Composition of *Aspergillus* section *Flavi* fungi in maize and groundnuts

Communities of *Aspergillus* section *Flavi* on maize consisted mostly of *A. flavus* (Table 1), while those on groundnut were dominated by *A. parasiticus* (Table 2). *Aspergillus flavus* L strain morphotype is reported to be the most prevalent member of *Aspergillus* section *Flavi* in soils and crops from many areas including maize, groundnut, cottonseed, rice, sorghum and almonds (Donner et al., 2015, Purcell et al., 1980, Schroeder and Boller, 1973). *Aspergillus flavus* is also a much more aggressive colonizer than *A. parasiticus* on groundnut (Horn, 2005). The current study stands in contrast to the aforementioned reports in that *A. parasiticus* occurred in frequencies higher than *A. flavus* in groundnut and at levels higher than has been observed on maize in East (Probst et al., 2007, Probst et al., 2010) or West Africa (Atehnkeng et al., 2008). The agroecologies in Zambia apparently differ from previously examined systems. This may reflect in part the percent of land in Zambia not in crop production. Indeed in agroecology I, the *A. flavus* L strain morphotype has displaced *A. parasiticus* in cultivated but not in non-cultivated soils (Table 5). As agriculture becomes more intensive, this trend may become more widespread.

### 4.2 Association between quantity of *Aspergillus* section *Flavi* and aflatoxin concentration

To assess the risk that a given fungal group poses to aflatoxin contamination of a crop, the aflatoxin-producing potential and frequency of occurrence of the fungus in contaminated crops need to be considered (Mehl et al., 2012, Probst et al., 2007). Regression analyses were conducted to examine agents associated with actual contamination events in market places previously reported in maize and groundnut in Zambia (Kachapulula et al., 2017). Percentages of the *Aspergillus* section *Flavi* community composed of the L strain *A. flavus* were inversely related to aflatoxin concentrations in groundnut in agroecology I (Table 4). This suggests that other components of these communities are more important causal agents. Increases in S-morphology fungi positively explained aflatoxin concentrations only in maize from agroecology II, indicating the potential of these fungi to exacerbate contamination. However, this was observed in only one area. Prevalence of *A. parasiticus* positively explained aflatoxin concentrations in groundnut in all agroecologies and in maize from agroecology III (Table 4). Atoxigenic *A. parasiticus* are rare (Horn et al., 1996). All *A. parasiticus* isolates in the current study were highly toxigenic (Table 7). High toxigenicity combined with high prevalence in infected crops suggests that *A. parasiticus* is the most important etiologic agent of aflatoxin contamination in Zambia. This was further supported by presence of both B & G aflatoxins in 5 highly contaminated maize and groundnut samples from markets.

### 4.3 *Aspergillus* section *Flavi* from cultivated and non-cultivated soils

Incidence of *A. parasiticus* in soils of Zambia were much higher than incidences previously observed in soils from East or West Africa (Donner et al., 2009). Natural dominance of *A. parasiticus* in the soil could contribute to crop from Zambia having higher levels of this species than has been observed elsewhere (Donner et al., 2009, Horn and Dorner, 1998). Horn and Dorner (1998) observed that *A. parasiticus* incidences were higher in fields cultivated to peanuts than in those where maize was grown. Most of the small scale farms sampled in the current study had mixed cropping, where maize or other cereals were grown in combination with groundnut (Tembo and Sitko, 2013). Wide cultivation of groundnut in agroecologies of Zambia (Sitko et al., 2011) may contribute to high incidences of *A. parasiticus* in the soil and eventually on the crop. Within the agroecologies of Zambia, *A. parasiticus* incidences were highest in agroecology III and lowest in I (Table 5). Soils in the three regions differ in pH and temperature, with region III being the coolest and most acidic (Bunyolo et al., 1995). Low temperature promotes crop colonization by *A. parasiticus* (Donner et al., 2015, Horn and Dorner, 1998) and could contribute to the higher frequencies of this fungus observed in agroecology III of Zambia (Table 5). However, perhaps the most important factor influencing incidences of *A. parasiticus* in crops in Zambia is the natural distribution of this species as reflected in composition of the fungal community in non-cultivated soils.

Fungi are capable of dispersal under natural conditions and mixtures of different aflatoxigenic and atoxigenic fungi are found in both non-cultivated and cultivated fields (Boyd and Cotty, 2001). In Zambia, many small-scale farmers grow their crops adjacent to national forests. The current study reports similar patterns of community composition in non-cultivated and cultivated areas (Table 6) suggesting that *Aspergillus* species endemic to non-cultivated areas in Zambia influence compositions of fungal communities in cultivated areas to a greater extent than *Aspergillus* introduced with crops. Application of atoxigenic *A. flavus* based biocontrol products may prevent this movement and associated crop contamination.

### 4.4 Aflatoxin production by *A. parasiticus* from crops and soils

Assessing the risk a given fungal group poses to aflatoxin contamination requires knowledge of the aflatoxin-producing potential and frequency of occurrence of the fungus (Cotty et al., 2008, Probst et al., 2007). High frequencies of *A. parasiticus* were observed in crops and soils from Zambia (Table 1, Table 2, Table 3, Table 5, Table 6) and were associated with contaminated crops (Table 4). *Aspergillus parasiticus* from Zambia was highly toxigenic (Table 7) irrespective of the fungus source (i.e. maize, groundnut, or soil), substrate used for assaying aflatoxigenicity (maize or groundnut) or temperature at which aflatoxigenicity assessments were conducted. *Aspergillus parasiticus* from non-cultivated areas was just as aflatoxigenic as those from crops or cultivated soil, suggesting that fungi endemic to non-cultivated areas in Zambia are potential reservoirs from which aflatoxigenic fungi disperse to crops. Similar to what has been reported before where aflatoxigenicity of *A. parasiticus* was the same on maize and groundnut at 31 °C (Kachapulula et al., 2017), no differences were observed on the two crops at 25 °C, 30 °C or 35 °C (Table 7). However, higher concentrations of aflatoxins were produced on groundnut than maize at 20 °C. The mechanisms behind higher aflatoxigenicity on groundnut than maize at lower temperatures are currently unknown.

Aflatoxin-producing fungi are common in maize and groundnut, and in cultivated and non-cultivated soil in all agroecologies of Zambia. *Aspergillus parasiticus* and fungi with S strain morphology are important etiologic agents of crop contamination*. Aspergillus flavus* L strain morphotype fungi were associated with lower aflatoxins in crops, possibly because this group of aflatoxin-producers are of lower average aflatoxin-production potential in Zambia (Kachapulula et al., 2017). L strain isolates with low aflatoxin-producing potential are also known to interfere with crop contamination by highly toxigenic fungi (Atehnkeng et al., 2014). Methods to increase incidences of atoxigenic L strain isolates, such as biocontrol (Atehnkeng et al., 2016), may lower aflatoxin contamination in Zambia. Cultivated and non-cultivated areas had comparable community structures of *Aspergillus* section *Flavi*. Moreover, fungi from non-cultivated areas were just as toxigenic as those from cultivated areas. Fungi endemic to non-cultivated areas in Zambia may influence the compositions of fungal communities in cultivated areas and are potential reservoirs from which toxigenic fungi disperse to crops. Treatments with atoxigenic genotypes of the *A. flavus* L strain morphotype may reduce effects of fungal communities from non-cultivated areas on fungi infecting crops.
